# Analysis of Healthcare Costs Incurred in Regional Hospitals in Andalusia (Spain) during the COVID-19 Pandemic

**DOI:** 10.3390/ijerph192316132

**Published:** 2022-12-02

**Authors:** Antonio Lopez-Villegas, Rafael Jesus Bautista-Mesa, Pedro Acosta-Robles, David Hidalgo-Serrano, Francisco Javier Aguirre-Ortega, Miguel Angel Castellano-Ortega, Maria Marta Mollo, Cesar Leal-Costa, Salvador Peiro

**Affiliations:** 1 Laboratory for Research, Education and Planning in Critical and Intensive Care Medicine, CTS-609 Research Group, Poniente University Hospital, 04700 El Ejido, Spain; 2 Economic-Financial Directorate, Alto Guadalquivir Health Agency, 23740 Andujar, Spain; 3 Management Direction, Poniente University Hospital, 04700 El Ejido, Spain; 4 Economic Directorate and General Services, Poniente University Hospital, 04700 El Ejido, Spain; 5 Information Technology Unit, Poniente University Hospital, 04700 El Ejido, Spain; 6 Medical Deputy, Alto Guadalquivir Health Agency, 23740 Andujar, Spain; 7 Management Control Unit, Alto Guadalquivir Health Agency, 23740 Andujar, Spain; 8 Nursing Department, University of Murcia, El Palmar, 30120 Murcia, Spain; 9 Health Services Research Unit, Foundation for the Promotion of Health and Biomedical Research of Valencia Region (FISABIO), 46020 Valencia, Spain

**Keywords:** cost analysis, COVID-19, healthcare, hospital emergency service, hospitalisation, intensive care unit, regional hospitals, Spain

## Abstract

(1) Background: The global health crisis caused by the coronavirus disease (COVID-19) pandemic has led to extreme overloading of different public healthcare systems worldwide. The Spanish Public Healthcare System is one of them. This study aimed to conduct a comparative cost analysis to assess the impact of the COVID-19 pandemic on small- and medium-sized regional hospitals in Andalusia (Spain). (2) Methods: This comparative, multicentre, observational, and retrospective study was designed to perform a comparative cost analysis between the Alto Guadalquivir Health Agency (AGHA) and Poniente University Hospital (PUH), both of which belong to the Spanish Public Health System (PHS). The data included in this study corresponds to the total costs by area and year incurred by the 61,335 patients from both healthcare institutions (AGHA = 36,110; PUH = 25,225) in the areas of hospital emergency service (HES), hospitalisation, and intensive care unit (ICU), during the 24 months of the study period (from 1 January 2019 to 31 December 2020). (3) Results: The analysis results showed a significant increase in costs incurred in 2020 for HES relative to those incurred in 2019 for both AGHA (+14%; *p* < 0.003) and PUH (+36%; *p* = 0.002). Additionally, costs incurred for ICU increased significantly in 2020 relative to those incurred in 2019 for both AGHA (+30%; *p* = 0.003) and PUH (+46%; *p* = 0.002). Hospitalisation costs for AGHA also increased significantly (+9%; *p* < 0.012) in 2020 versus those obtained in 2019; however, no significant differences were found for PUH (+6%; *p* = 1) in the same period analysed. However, the number of patients treated in the areas of HES, hospitalisation, and ICU was significantly reduced throughout 2020 compared to 2019. (4) Conclusions: Our findings show that the costs incurred during 2020 in the regional hospitals of Andalusia (Spain) increased significantly in most of the parameters analysed relative to those incurred in the year before the pandemic (i.e., 2019).

## 1. Introduction

Since the Chinese health authorities notified the World Health Organization (WHO) about the first case of an unknown type of pneumonia more than 2 years ago, the lives of millions of people worldwide have changed dramatically [1,2,3,4]. Within a short period, coronavirus disease (COVID-19), caused by severe acute respiratory syndrome coronavirus 2 (SARS-CoV-2), had spread rapidly across the world, thus triggering the first pandemic of this century [5,6,7]. With the emergence of the Omicron variant, the number of infections increased significantly as this variant has the ability to replicate and spread at a much faster rate than the original virus causing COVID-19 and the Delta variant. According to experts from The Centers for Disease Control and Prevention, a person infected with Omicron can spread the virus to others regardless of their vaccination status or the presence of symptoms [8]. To date, 614,385,693 confirmed cases of COVID-19 have been reported worldwide, including 6,522,600 deaths, reported to the WHO [9]. However, the true number of infected patients and deaths remains unknown [10].

Spain’s first SARS-CoV-2 case was reported on 30 January 2020 [11]. Since then, the number of infected cases in Spain, as well as the number of associated deaths (114,179), has continued to increase (13,422,984) [12,13,14]. The global health crisis caused by the COVID-19 pandemic has led to extreme overloading of different public healthcare systems worldwide, and a distinct one among them is the Spanish healthcare system [15,16,17,18].

Since the emergence of COVID-19, a multitude of studies related to different aspects of this disease have been conducted [19,20]. However, the social and economic repercussions of COVID-19 are more severe than those caused by the economic crisis after the Great Depression in the 1930s or the Second World War [10]. Despite more than 2 years having passed since the start of the health crisis, the economic and social levels reached in December 2019 have still not been achieved [21,22,23]. Although research efforts are being made extensively in this field, only a limited number of studies describing the economic impact of this disease are available [4,24,25,26,27,28]. There is considerable heterogeneity across costing methodologies (simulation, absolute costs, etc.). We observed a great variation in costs per patient, making comparison challenging even among countries with similar economic status [26,27,28]. However, no studies have performed a comparative analysis of ‘intra-hospital’ costs, especially for the costs incurred in important areas such as the emergency department, hospitalisation, and intensive care unit of the same hospital, between 2019 (pre-pandemic) and 2020 (first year of the pandemic). In addition, no studies have reported a comparative analysis of costs by area and type of costs between different small- and medium-sized regional hospitals (inter-hospital comparison). There is an urgent need for a study focusing on this aspect, which may be of interest in the management of future outbreaks [7,29,30]. We conducted a study wherein the main objective was to perform a cost analysis to assess the impact of the COVID-19 pandemic on small- and medium-sized regional hospitals of Andalusia (Spain).

## 2. Materials and Methods

### 2.1. Design and Setting

This comparative, multicentre, observational, and retrospective study was designed to perform a comparative analysis of costs in small- and medium-sized regional hospitals located in central and eastern Andalusia (Spain).

The 9 healthcare centres participating in this study were as follows: (1) Alto Guadalquivir Health Agency is responsible for healthcare management in the provinces of Jaen and Cordoba (Andalusia, Spain). It serves a reference population of 238,856 inhabitants, distributed in 8 healthcare centres, as follows: 62,128 correspond to the Alto Guadalquivir Hospital in Andújar, 61,687 to the Montilla Hospital, 24,114 to the Sierra de Segura High Resolution Hospital, 30,048 to the Puente Genil High Resolution Hospital, 10,498 to the Alcaudete High Resolution Hospital, 23,143 to the Valle del Guadiato High Resolution Hospital, 23,143 to the Alcalá la Real High Resolution Hospital, and 27,238 to the Sierra de Cazorla High Resolution Hospital [31]. (2) The Poniente University Hospital, located in the province of Almería (Andalusia, Spain), has a reference population of 265,153 inhabitants [32]. Table 1 shows the main characteristics of both healthcare institutions (year 2019).

### 2.2. Cost Analysis

Between October 2020 and June 2021, total costs by area and year incurred by the 61,335 patients from both healthcare institutions (AGHA = 36,110; PUH = 25,225) in the areas of hospital emergency service (HES), hospitalisation, and intensive care unit (ICU) during the 24 months of the study period (from 1 January 2019 to 31 December 2020) were included. The data on the generated economic costs were obtained from the Economic Management Units of both healthcare institutions.

Direct costs from the hospital perspective were calculated in euros (year 2020). Both healthcare institutions belong to the Spanish Public Health System. Discounts and indirect costs were not considered in the calculation. Total costs by area and year in the areas of hospitalisation, intensive care unit, and hospital emergency services (HES) were included in this study. The costs of laboratory tests (biotechnology), maintenance/cleaning, human resources, material resources, and imaging studies (radiography) utilised during the stay of patients in the previously mentioned areas were also accounted such as ’type of cost and year’. Indirect costs were not included in the analysis because it requires imputation techniques, which could distort the comparison between different hospitals (in addition to the difficulty in obtaining this information).

### 2.3. Ethical Considerations

The study protocol was approved by the Regional Ethics Committee for Health Research (CEIC-AL: 91/2020). The present study was conducted under the precepts of the Declaration of Helsinki [33] and Spanish laws on data protection and patient rights [34,35].

### 2.4. Statistical Analysis

First, a descriptive analysis was conducted. The cost variable was considered as a quantitative variable. Therefore, measures of central tendency (mean, median, and confidence interval for the mean) were calculated by month and for the entire year. The normality of the quantitative variable was tested with the Kolmogorov–Smirnov test. To test the hypothesis of equality of location measures for different intra-hospital years, Student’s *t*-test was used for dependent samples with a normal distribution; otherwise, the Wilcoxon signed-rank test was used. For a comparison between the healthcare institutions, Student’s *t*-test was used for independent samples with a normal distribution; otherwise, the Mann–Whitney U test was used. *p*-value was estimated using the Wilcoxon signed-rank test for dependent samples comparing both institutions for the two years. Similarly, nonparametric tests were performed to compare the type of cost in both healthcare institutions for the two years. The Bonferroni correction method was used to control the ‘overall significance level’ of the set of comparisons performed. All statistical analyses were performed using the free software program R version 4.0.5 (Vienna, Austria, https://www.r-project.org/, accessed on 15 May 2022); *p* ≤ 0.05 was considered to indicate statistical significance.

## 3. Results

### 3.1. Descriptive Analysis of Costs Analysed by Area and Year Carried out for Both Healthcare Institutions before and during the First Year of the Pandemic

**Figure 1:**
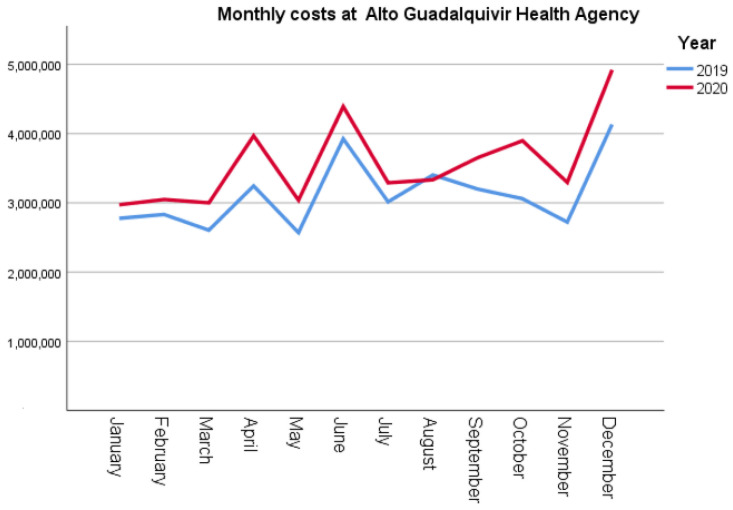
Comparative analysis of monthly costs at Alto Guadalquivir Health Agency.

The costs of hospitalisation in AGHA increased significantly (*p* = 0.012) in 2020 relative to 2019 (Table 2). As for the intensive care unit, the costs increased by EUR 443,367 in 2020, which represents a 30% increase (*p* = 0.003), compared to 2019. Moreover, emergency service costs were 14% higher than those incurred in the year before the pandemic, that is, in 2019 (*p* = 0.003). As shown in Figure 1, the average monthly costs in 2020 were higher than those in 2019, except in August.

**Figure 2:**
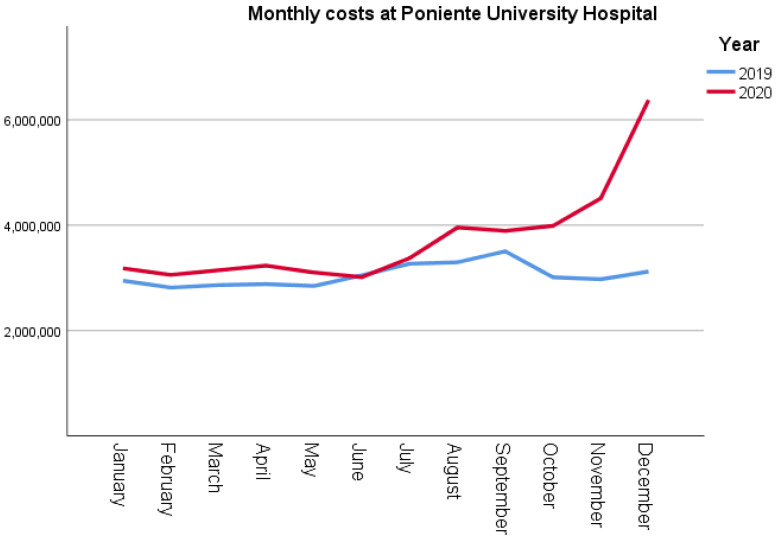
Comparative analysis of monthly costs at Poniente University Hospital.

The results of the analysis of the data collected from PUH (Table 2) showed that the number of patients treated in 2020, was significantly reduced in the three areas, except in the intensive care unit (ICU). Despite an increase of EUR 91,098 in the costs associated with hospitalisation, growing by 6% in the previous year, there was no significant difference between the two costs. In 2020, the costs incurred for the intensive care unit increased by 46% (*p* = 0.002) relative to those in 2019. Additionally, emergency service costs were 36% higher in 2020 than in 2019. As shown in Figure 2, monthly costs in PUH started to increase significantly, relative to those in 2019, from October in 2020.

### 3.2. Descriptive Analysis of Costs Analysed by Type of Cost and Year

**Figure 3:**
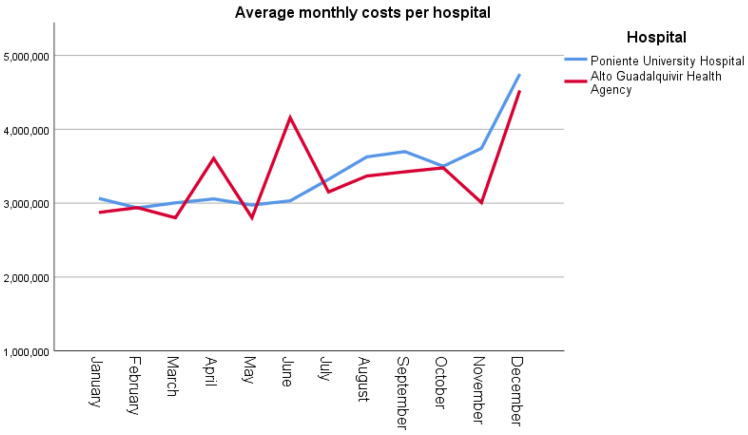
Average monthly costs incurred in both regional hospitals.

In AGHA, a significant increase was noted in costs associated with most of the parameters in 2020 relative to those obtained in 2019 (Table 3). The economic impact moved in a range between 12% for human resources (*p* = 0.003), passing through 21% for X-ray (*p* = 0.006), 23% for material resources (*p* = 0.05), and finally 33% (*p* = 0.003) for biotechnology. On the other hand, despite having increased costs by EUR 1993, there were no significant differences with respect to the previous year. As shown in Figure 3, the average monthly costs were higher in AGHA in the second quarter of 2020 than those in PUH, a finding coinciding with that observed in the first wave of the COVID-19 pandemic.

In the case of PUH, there was a very significant increase in costs associated with most of the variables in 2020 relative to those obtained in 2019 (Table 3). The increase in costs moved in a range that fluctuated from 12% reached in human resources (*p* = 0.008), passing through 67% in biotechnology (*p* = 0.008), with 98% reached in material resources (*p* = 0.005) to, finally, a very significant growth of 127% (*p* = 0.005) reached in maintenance/cleaning. However, despite the 18% increase in costs for radio-diagnostic tests (radiography), no significant differences were noted when compared with the costs incurred in 2019 (*p* = 0.06).

### 3.3. Cost Comparison between AGHA and PUH

Table 4 shows a comparison of the costs incurred for the different areas and type of cost between AGHA and PUH for 2020. The results showed significant differences between the two healthcare institutions in the areas of hospital emergency service and hospitalisation (*p* = 0.000). As for the type of cost, significant differences were noted in all the parameters except in the costs incurred for human resources (*p* = 0.821). As shown in Figure 3, the average monthly costs were higher in AGHA in the second quarter of 2020 than in PUH, a finding coinciding with that observed in the first wave of the COVID-19 pandemic.

**Figure 4:**
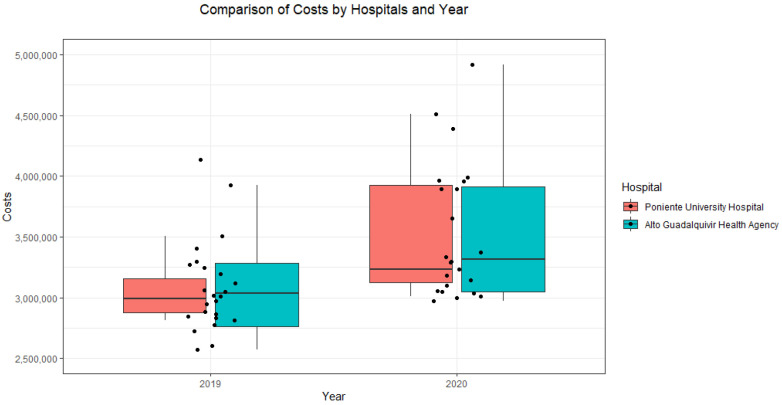
Comparison of costs by year between the regional hospitals.

As shown in Figure 4, barely any difference was noted in ‘total costs’ between the two hospitals, but the data showed a clear difference for both years, with an increase in costs in 2020.

## 4. Discussion

### 4.1. Main Findings

To the best of our knowledge, this has been the first multicentre study to present the results of a comparative analysis performed with the main objective of quantifying the increase in costs, in general terms, associated with the emergence of the pandemic caused by COVID-19 in small- and medium-sized regional hospitals (AGHA and PUH) in Andalusia (Spain).

The comparative analysis of costs showed that in 2020 (relative to 2019), there was a significant increase in the average costs in the three areas analysed (hospitalisation, intensive care unit, and HES), except in hospitalisation of PUH in that, although the average costs increased by EUR 91,098, they were not significant.

As for the average annual number of patients attended, a significant reduction was noted in both hospitals. The reduction was significant in the emergency departments of both hospitals. The number of hospitalised patients was found to be reduced in both centres, with the reduction being significant only in PUH. The average number of patients admitted to the ICU was significantly reduced in AGHA in 2020. In contrast, in the data obtained for PUH, the average number of ICU patients increased in 2020 compared with that observed in 2019; however, this increase was not significant.

The mean total cost per patient increased significantly in the three analised areas of all the centres participating in this study. The mean bill amount per hospitalised patient (AGHA = EUR 905.26 versus PUH = EUR 1605.38) increased significantly in both institutions (no distinction is madebetween patients with or without COVID-19); this value was lower than that reported in literature in previous studies on COVID-19 patients [36,37,38]. Although, in general terms, the average costs per patient and days of stay in the hospitalisation unit and ICU in 2020 increased significantly in the two centres, a significant increase was found only in the costs associated with hospitalisation in PUH. The average cost per patient for each day of stay in the ICU (including all types of pathologies) in both centres (ASAG = EUR 4633.27 versus HUP = EUR 6117.54) was higher than that reported in previous studies (focused on the economic analysis of COVID-19 patients) conducted in India ($3192.06) [36], South Africa (EUR 541.60) [39], Turkey (EUR 2924) [40], Iran, (EUR 3755) [41] and significantly lower than the results shown in studies performed in Saudi Arabia (EUR 7316) [42] and the United States (EUR 95,546–EUR 301.331) [43].

As for the comparative analysis conducted according to type of cost and year, a generalised increase was seen in the average annual costs in all the parameters evaluated. The increase in average costs was significant in the areas of biotechnology, human resources, and material resources in both hospitals. As for maintenance/cleaning, the increase in average costs per year was significant only in PUH. In contrast, the average annual costs associated with the performance diagnostic tests (radiography) showed a significant increase only in AGHA.

The average costs by area were higher for hospitalisation (AGHA: +9% versus PUH: +6%) and ICU in PUH (+46%) than those for AGHA (30%). However, the average costs for the emergency departments were higher in AGHA than in PUH. The differences in the average costs per area between both medical hospitals were significant in the hospitalisation and emergency services areas.

A comparison of the average costs by type of cost showed that the costs were higher for human resources, material resources, and diagnostic tests (radiography) in AGHA, with significant differences in the last two parameters, than those in PUH. In PUH, the average costs were higher for biotechnology and maintenance/cleaning, with significant differences in both hospitals when compared with those in AGHA.

### 4.2. Limitations and Strengths

This comparative analysis of healthcare activity had some methodological weaknesses and strengths that need to be taken into account.

First, the data provided by both PUH and AGHA were not disaggregated; hence, it was not possible to perform a comparative economic analysis among patients diagnosed with COVID-19. This limitation indicates that the results presented in this study could not be compared with those of previous studies because most of the available publications have shown results centred on COVID-19 patients.

Second, the costs associated with areas other than hospitalisation, ICU, and emergency care, such as pharmacy, were not included in the analysis, which limits the generalisability of the study results.

Third, this study was conducted from the perspective of the Andalusian Public Health System, belonging to the Spanish Public Health System. For this reason, it was not possible to perform an analysis of indirect costs from patients’ perspective, and it was not possible to estimate informal costs such as travel expenses and/or loss of work productivity of patients and/or accompanying persons, because it was not possible to interview the patients. Therefore, the economic impact would have been very different.

The fourth limitation and coinciding with the finding of a previous study [44] is the retrospective nature of the study design and the relatively small- and/or medium- size of the selected health centres. This aspect reduces the possibility of extrapolating the study results to other hospital centres. Furthermore, the results presented are directly related to the incidence of COVID-19 in a specific period of time.

The strengths of this study are directly related to the description of the comparative economic analysis performed. The study provides relevant information on the significant increase in the budget attributed to hospital care activity as a consequence of the emergence of COVID-19 in small- and/or medium-sized regional hospitals in Andalusia (Spain) when compared with that in the year before the pandemic. In addition, the Bonferroni correction method was used to control the ‘overall significance level’ of the set of comparisons performed.

These results can be used by public health service managers to further investigate the clinical impact of COVID-19 in areas not included in this study. Additionally, future cost–opportunity studies could reveal the indirect costs borne by patients and public healthcare systems owing to the reduction in hospital care activity caused by COVID-19.

## 5. Conclusions

The results of this study show that the costs incurred during 2020 in the regional hospitals of Andalusia (Spain) increased significantly in most of the parameters analysed relative to those in the year before the pandemic. The number of patients treated in HES, hospitalisation, and ICU was found to be significantly reduced during the first year of the pandemic. Additionally, the periods in which there was a greater increase in expenditure coincided with the peak periods of the first wave (March–June) and the second wave (in the last quarter of the year) of COVID-19 in Spain.

## Figures and Tables

**Table 1:** Main characteristics of the regional hospitals included in the study (year 2019).

	Alto Guadalquivir Health Agency [31]	Poniente University Hospital [32]
Reference population (inhabitants)	238,856	265,153
Municipalities covered	40	15
Total professionals working	1946	1710
Outpatient Consultations	346,762	229,284
Emergencies	262,021	175,649
Surgical activity	11,533	13,029
Admissions	8686	13,878
Deliveries	921	2593
Laboratory diagnostic tests	5,719,469	4,183,740
Diagnostic radiology tests	278,896	182,255
Healthcare centres included	8	1

**Table 2:** Descriptive statistics of costs by area and year for both periods.

Inputs	Alto Guadalquivir Health Agency			Poniente University Hospital		
	2019	2020	*p*-Value	2019	2020	*p*-Value
Mean Costs per Area and Year						
Hospitalisation * [(mean) (95 CI)	590,397 (542,762; 638,032)	642,648 (585,477; 699,819)	<0.05	1,452,965 (1,388,348; 1,517,582)	1,544,063 (1,301,394; 1,786,731)	1
ICU * (mean) (95 CI)	282,780 (247,276; 318,284)	367,254 (320,140; 414,369)	<0.01	290,026 (271,961; 308,090)	422,886 (330,144; 515,628)	<0.01
Emergencies * (mean) (95 CI)	2,250,119 (2,009,935; 2,490,302)	2,556,760 (2,260,784; 2,852,736)	<0.01	1,305,332 (1,241,810; 1,368,853)	1,768,957 (1,473,247; 2,064,667)	<0.01
Patients treated per year						
Emergencies (95 CI)	21,055 (19,784.06; 22,326.44)	13,512 (10,089.96; 16,934.04)	<0.01	13811 (13,320.15; 14,302.52)	9255 (6664.52; 11,847.31)	<0.01
Hospitalisation (95 CI)	762 (716.97; 807.86)	725 (658.20; 792.14)	0.24	1128 (1079.97; 1177.53)	981 (888.20; 1074.80)	<0.01
ICU (mean) (95 CI)	31 (19.44; 35.76)	24 (19.44; 28.72)	<0.01	21 (18.74; 24.43)	25 (21.28; 29.05)	0.10
Total (95 CI)	21,849 (20,542.32; 23,155.34)	14,261 (10,780.16; 17,742.34)	<0.01	14,962 (14,441.87; 15,481.47)	10,263 (7584.45; 12,940.72)	<0.01
Mean cost per patient and year						
Emergencies (95 CI)	107.71 (95.06; 120.37)	226.01 (146.08; 305.93)	<0.01	94.80 (88.96; 100.63)	229.93 (154.97; 304.89)	<0.01
Hospitalisation (95 CI)	783.00 (695.41; 870.60)	905.26 (783.08; 1,027.44)	<0.01	1296.75 (1189.00; 1404.50)	1605.38 (1313.48; 1897.27)	0.05
ICU (mean) (95 CI)	9357.31 (8111.10; 10,603.52)	17,204.93 (11,470.47; 22,939.39)	<0.01	13,770.68 (12,492.18; 15,049.18)	17,595.65 (13,391.28; 21,800.03)	0.05
Total (95 CI)	144.13 (127.67; 160.58)	294.30 (195.53; 393.06)	<0.01	204.50 (191.38; 217.63)	427.63 (294.47; 560.79)	<0.01
Mean cost per patient and stay day (night included)						
Hospitalisation (95 CI)	199.04 (173.71; 224.38)	207.88 (182.63; 233.13)	0.27	227.71 (204.30; 251.12)	275.76 (231.73; 319.78)	0.02
ICU (mean) (95 CI)	3165.26 (2460.01; 3870.51)	4633.27 (3072.23; 6194.32)	0.14	3713.85 (2184.44; 5243.27)	6117.54 (3457.37; 8777.71)	0.06
Total (95 CI)	1023.00 (868.14; 1177.86)	1120.62 (968.50; 1272.74)	<0.05	470.03 (423.56; 516.50)	655.49 (551.15; 759.82)	<0.01

CI = confidence interval; ICU = intensive care unit; * all amounts are expressed in EUR.

**Table 3:** Descriptive statistics of costs by type of mean cost and year for both periods.

Type of Cost	Alto Guadalquivir Health Agency			Poniente University Hospital		
	2019	2020	*p*-Value	2019	2020	*p*-Value
Biotechnology (mean) (95 CI)	168,719 (153,603; 183,834)	224,105 (192,739; 255,470)	0.003	211,337 (187,345; 235,328)	352,514 (250,406; 454,621)	0.008
Maintenance/Cleaning (mean) (95 CI)	90,981 (87,750; 94,211)	92,973 (87,464; 98,482)	0.814	118,088 (113,562; 122,614)	268,098 (−32,839; 569,036)	0.005
Human Resources (mean) (95 CI)	2,552,350 (2,265,722; 2,838,979)	2,869,871 (2,532,836; 3,206,906)	0.003	2,519,458 (2,396,733; 2,642,183)	2,819,940 (2,588,181; 3,051,699)	0.008
Material Resources (mean) (95 CI)	131,780 (116,382; 147,179)	161,862 (137,747; 185,977)	0.05	75,345 (63,678; 87,013)	149,480 (108,330; 190,629)	0.005
X-rays (mean) (95 CI)	179,466 (154,519, 204,414)	217,853 (190,498; 245,208)	0.006	124,093 (115,636; 132,551)	145,874 (111,764; 179,984)	0.06

All amounts are expressed in EUR.

**Table 4:** Comparison of costs by year incurred by area and type of cost between the two regional hospitals.

	Alto Guadalquivir Health Agency	Poniente University Hospital	*p*-Value
Area			
Hospitalisation * (mean) (95 CI)	616,522 (580,512; 652,533)	1,498,514 (1,381,434; 1,615,593)	0.00
ICU * (mean) (95 CI)	325,017 (292,350; 357,684)	356,456 (304,428; 408,484)	0.536
Emergencies * (mean) (95 CI)	2,403,439 (2,216,183; 2,590,696)	1,537,144 (1,365,906; 1,708,382)	0.00
Type of cost			
Biotechnology * (mean) (95 CI)	196,412 (176,442; 216,381)	281,925 (224,907; 338,943)	0.003
Maintenance/Cleaning * (mean) (95 CI)	91,977 (89,010, 94,943)	193,093 (51,031; 335,155)	0.000
Human Resources * (mean) (95 CI)	2,711,111 (2,496,542; 2,925,679)	2,669,699 (2,532,851; 2,806,548)	0.821
Material Resources * (mean) (95 CI)	146,821 (132,157; 161,485)	112,412 (87,073; 137,752)	0.001
X-rays * (mean) (95 CI)	198,660 (179,737; 217,583)	134,984 (118,163; 151,805)	0.000

ICU = intensive care unit; * All amounts are expressed in EUR.

## Data Availability

The datasets used and/or analysed in the current study are available from the corresponding author upon request.
